# Improving inference for nonlinear state‐space models of animal population dynamics given biased sequential life stage data

**DOI:** 10.1111/biom.13267

**Published:** 2020-04-25

**Authors:** Leo Polansky, Ken B. Newman, Lara Mitchell

**Affiliations:** ^1^ U.S. Fish and Wildlife Service Bay‐Delta Field Office Sacramento California; ^2^ U.S. Fish and Wildlife Service Lodi Field Office Lodi California; ^3^ Biomathematics & Statistics Scotland and School of Mathematics The University of Edinburgh Edinburgh UK

**Keywords:** Bayesian hierarchical models, data integration, delta smelt, *Hypomesus transpacificus*, parameter identifiability, San Francisco Estuary

## Abstract

State‐space models (SSMs) are a popular tool for modeling animal abundances. Inference difficulties for simple linear SSMs are well known, particularly in relation to simultaneous estimation of process and observation variances. Several remedies to overcome estimation problems have been studied for relatively simple SSMs, but whether these challenges and proposed remedies apply for nonlinear stage‐structured SSMs, an important class of ecological models, is less well understood. Here we identify improvements for inference about nonlinear stage‐structured SSMs fit with biased sequential life stage data. Theoretical analyses indicate parameter identifiability requires covariates in the state processes. Simulation studies show that plugging in externally estimated observation variances, as opposed to jointly estimating them with other parameters, reduces bias and standard error of estimates. In contrast to previous results for simple linear SSMs, strong confounding between jointly estimated process and observation variance parameters was not found in the models explored here. However, when observation variance was also estimated in the motivating case study, the resulting process variance estimates were implausibly low (near‐zero). As SSMs are used in increasingly complex ways, understanding when inference can be expected to be successful, and what aids it, becomes more important. Our study illustrates (a) the need for relevant process covariates and (b) the benefits of using externally estimated observation variances for inference about nonlinear stage‐structured SSMs.

## 1 INTRODUCTION

Understanding what determines changes in animal abundance through time is a basic question of population ecology and natural resource management. Estimates of recruitment and survival can facilitate this, quantifying where, when, and how populations respond to endogenous (density‐dependent) and exogeneous (external, density‐independent) factors (Turchin, 2003). However, fitting models can be difficult in practice because field‐based estimates of animal population abundances can be incorrect due to sample randomness as well as systematic biases (Staudenmayer and Buonaccorsi, 2006). Although population models addressing observation error due to sampling variability are increasingly common, the inclusion of observation bias has been much less studied because most analyses use annual time step models of data based on a single survey and the bias is assumed to cancel out. Relative biases can be especially problematic when abundances from different surveys are integrated into a single population model.

State‐space models (SSMs) offer an attractive framework to integrate disparate datasets with potentially different biases. SSMs are proving to be important population modeling tools (Newman *et al*., 2014) because they allow separate description of process and observation time series. The state process time series consists of unobserved values that may be viewed as an underlying description of the true, alternatively latent or hidden (Newman *et al*., 2006), state of a dynamic system. The observation time series consists of measurements on the state process. The explicit separation of process variation from observation error allows the flexibility needed to address survey specific errors.

One problem long recognized with some SSMs, especially the special case of normal dynamic linear models (NDLMs; West and Harrison, 2006), is the difficulty of simultaneously estimating both process variance and observation variance (Dennis *et al*., 2006; Knape, 2008; Knape *et al*., 2011; Auger‐Méthé *et al*., 2016). There are similar difficulties in jointly estimating process variability and observation noise variances in nonlinear population dynamics process models (de Valpine and Hilborn, 2005).

Several remedies for SSM inference have been explored. One is to formulate the SSM such that the parameters of the process variance are also parameters for the mean(s) of the state process. Examples of such parameterizations with NDLMs include Newman (1998) who used normal approximations for binomial and multinomial distributions where the expectations and variances matched those distributions, and Besbeas *et al*. (2002) who approximated Poisson and binomial distributions with the corresponding means and variances. A second remedy is to collect replicate observations that allow separation of observation variance from process variance (Dennis *et al*., 2010). A third remedy is to avoid trying to estimate the observation variance altogether by inserting external estimates of observation variance when such estimates are available (Knape *et al*., 2013), for example, through mark‐recapture sampling.

With few exceptions, for example, Knape *et al*. (2013), most assessments of SSM identifiability problems have been based on NDLMs and often for situations where the observations are annual and based on one survey type. In this paper, we examine the estimation problem of an SSM whose state process is stage‐structured, nonlinear, and non‐Gaussian, and whose observations have stage‐specific relative biases. Stage‐structured population models are an important tool to draw inference about factors affecting recruitment and survival rates (Caswell, 2001). Although some examples of their application embedded within an SSM inferential framework exist (de Valpine, 2003; de Valpine and Rosenheim, 2008), the effects of both noisy and biased data on inference for nonlinear SSMs are relatively poorly understood. Additionally, in practice, ecologists are equally concerned with estimation of coefficients relating predictor variables to vital rates, also a topic of little focus compared to historical emphasis on estimation of variance parameters and latent states.

The rest of the paper is structured as follows. In Section 2, we develop a stage‐structured SSM that contains stochastic vital rates with covariate dependency and an observation model with bias terms for some life stages. We also describe several alternate formulations for the observation model depending on whether external estimates of observation variances are available. Section 3 provides a theoretical analysis of the identifiability of the parameters in this model while Section 4 presents a simulation study investigating parameter inference in practice. Attention is given to how estimation properties differ between models that fix observation error variance using externally derived values and models that internally estimate observation error variance. Section 5 presents a case study on the fish species delta smelt (*Hypomesus transpacificus*) to illustrate the utility of such models and highlight practical issues that arise when fitting them. Discussion is in Section 6.

## 2 MODEL DESCRIPTION

Throughout we will parameterize the lognormal distribution using $\text{LogNormal}(\mu ,\sigma^{2})$, where μ and σ^2^ are the log‐mean and log‐variance parameters, respectively. Similarly, logit‐normal distributions will be described using $\text{LogitNormal}(\mu ,\sigma^{2})$, where μ and σ^2^ are the location and squared‐scale parameters. The distribution designation of LogitNormal means that the logit transformed survival probabilities are normally distributed.

### 2.1 State process model

Assume a population can be partitioned into different life stages where $n_{s,t}$ denotes the true abundance of life stage *s* of time (or cohort) *t*. In particular, we consider a fish population that has four life stages: post‐larvae, juveniles, sub‐adults, and adults. Given an initial abundance of reproducing adults $n_{A,t=0}$ in cohort t=0, the state process update equations are

(1) $$\text{Post-larvae}|\text{Adults}:n_{PL,t}|n_{A,t-1}=\rho_{t}n_{A,t-1},$$

(2) $$\text{Juveniles}|\text{Post-larvae}:n_{J,t}|n_{PL,t}=\phi_{PL,t}n_{PL,t},$$

(3) $$\text{Sub-adults}|\text{Juveniles}:n_{SA,t}|n_{J,t}=\phi_{J,t}n_{J,t},$$

(4) $$\text{Adults}|\text{Sub-adults}:n_{A,t}|n_{SA,t}=\phi_{SA,t}n_{SA,t},$$

where $\rho_{t}$ is time‐specific recruitment and $\phi_{s,t}$ are the life stage and time‐specific survival probabilities. Assuming (environmental) stochasticity in four processes, the vital rate models for recruitment and the three survival probabilities are

(5) $$\text{Recruitment}:\rho_{t}∼\text{LogNormal}\left(x_{R,t}^{T}\zeta ,\sigma_{P,R}^{2}\right),$$

(6) $$\begin{array}{c} \text{Post-larval}\;\text{Survival}:\phi_{PL,t}∼\text{LogitNormal}\left(x_{PL,t}^{T}\beta ,\sigma_{P,PL}^{2}\right), \end{array}$$

(7) $$\text{Juvenile}\;\text{Survival}:\phi_{J,t}∼\text{LogitNormal}\left(x_{J,t}^{T}\eta ,\sigma_{P,J}^{2}\right),$$

(8) $$\begin{array}{c} \text{Sub-adult}\;\text{Survival}:\phi_{SA,t}∼\text{LogitNormal}\left(x_{SA,t}^{T}\gamma ,\sigma_{P,SA}^{2}\right), \end{array}$$

where $\zeta^{T}=\left(\zeta_{0},\ldots ,\zeta_{m_{R}}\right)$ is a vector of $m_{R}+1$ regression coefficients corresponding to a vector of recruitment predictor variables $x_{R,t}^{T}=\left(1,x_{1,t},\ldots ,x_{m_{R},t}\right)$, and $\sigma_{P,R}^{2}$ is the recruitment process variance on the log scale. The terms in the survival functions, Equations (6)‐(8), are defined analogously but with possibly different dimensions reflecting life stage‐specific numbers of covariates used in survival predictions.

### 2.2 Observation model

Observation error in the abundance estimates ${\hat{n}}_{s,t}$ can include both bias, such that $E\left[{\hat{n}}_{s,t}|n_{s,t}\right]=\psi_{s,t}n_{s,t}$, and sampling variance $V[{\hat{n}}_{s,t}]$ (Staudenmayer and Buonaccorsi, 2006). Different choices of the conditional distribution for ${\hat{n}}_{s,t}|n_{s,t}$ can be made, which in turn can affect inference (Knape *et al*., 2011). We chose a lognormal distribution because it ensures strictly positive abundance indices (especially useful when abundance indices are near zero), it is perhaps the most common assumption (although more often in terms of a normal distribution when working with log abundances), and because earlier work (Polansky *et al*., 2019) suggests it is to be preferred over a normal distribution for the case study model described in Section 5.

Due to nonidentifiability problems, we do not consider estimating biases that are both life stage and cohort specific because in that case the parameters would confound with the latent abundance estimates. Motivated by the case study in Section 5, we assume juvenile and sub‐adult abundance indices are biased by factors $\psi_{J}$ and $\psi_{SA}$, and we use the following parameterizations for the observation model equations:

(9) $${\hat{n}}_{PL,t}∼\text{LogNormal}\left(\ln\left(n_{PL,t}\right)-\frac{\sigma_{O,PL,t}^{2}}{2},\sigma_{O,PL,t}^{2}\right),$$

(10) $${\hat{n}}_{J,t}∼\text{LogNormal}\left(\ln\left(\psi_{J}n_{J,t}\right)-\frac{\sigma_{O,J,t}^{2}}{2},\sigma_{O,J,t}^{2}\right),$$

(11) $$\begin{array}{c} {\hat{n}}_{SA,t}∼\text{LogNormal}\left(\ln\left(\psi_{SA}n_{SA,t}\right)-\frac{\sigma_{O,SA,t}^{2}}{2},\sigma_{O,SA,t}^{2}\right), \end{array}$$

(12) $${\hat{n}}_{A,t}∼\text{LogNormal}\left(\ln\left(n_{A,t}\right)-\frac{\sigma_{O,A,t}^{2}}{2},\sigma_{O,A,t}^{2}\right).$$

With these parameterizations, the expected index is

(13) $$E\left[{\hat{n}}_{s,t}\right]=\left\{\begin{array}{cc} n_{s,t} & \text{observation}\;\text{bias}\;\text{absent}\\ \psi_{s}n_{s,t} & \text{observation}\;\text{bias}\;\text{present} \end{array}\right.$$

and the variance is

(14) $$\begin{array}{c} V\left[{\hat{n}}_{s,t}\right]=\left\{\begin{array}{cc} \left(e^{\sigma_{O,s,t}^{2}}-1\right)n_{s,t}^{2} & \text{observation}\;\text{bias}\;\text{absent}\\ \left(e^{\sigma_{O,s,t}^{2}}-1\right){\left(\psi_{s}n_{s,t}\right)}^{2} & \text{observation}\;\text{bias}\;\text{present}. \end{array}\right.\; \end{array}$$

Irrespective of whether a bias factor is included, the coefficient of variation (CV) is

(15) $$CV\left[{\hat{n}}_{s,t}\right]=\sqrt{e^{\sigma_{O,s,t}^{2}}-1}$$

from which it follows that

(16) $$\sigma_{O,s,t}^{2}=\ln\left(CV{\left[{\hat{n}}_{s,t}\right]}^{2}+1\right).$$

Plugging the right‐hand side of Equation (16) into Equations (9)‐(12), the observation model equations can be written as

(17) $$\begin{array}{ccc} {\hat{n}}_{s,t} & ∼ & \text{LogNormal}\left(\ln\left(\frac{\psi_{s}n_{s,t}}{\sqrt{CV{\left[{\hat{n}}_{s,t}\right]}^{2}+1}}\right),\ln\left(\mathrm{CV}{\left[{\hat{n}}_{s,t}\right]}^{2}1+1\right)\right), \end{array}$$

where $\psi_{s}=1$ if there is no bias term present in a given life stage or survey specific observation model.

### 2.3 Observation model formulations

There are three scenarios for how the observation model equations can be formulated. These scenarios are presented in Table 1. Scenarios 1 and 2 depend on the availability of estimates of uncertainty (either coefficients of variation or variances) for ${\hat{n}}_{s,t}$ that are derived *externally* to the SSM. In Scenario 3, observation variance is estimated within the SSM. The choice between Scenarios 1 and 2 may depend on whether estimated coefficients of variation or estimated variances are closer to the true values.

**TABLE 1:** Different scenarios for how observation model equations can be formulated, including cases where external estimates of observation error variance are available

Scenario	Equation and description
1	${\hat{n}}_{s,t}∼\text{LogNormal}\left(\ln\left(\frac{\psi_{s}n_{s,t}}{\sqrt{\hat{CV}{\left[{\hat{n}}_{s,t}\right]}_{Ex}^{2}+1}}\right),\ln\left(\hat{CV}{\left[{\hat{n}}_{s,t}\right]}_{Ex}^{2}+1\right)\right)$
	Use the external coefficient of variation estimates within Equation (17).
2	${\hat{n}}_{s,t}∼\text{LogNormal}\left(\ln\left(\frac{\psi_{s}n_{s,t}}{\sqrt{\hat{V}{\left[{\hat{n}}_{s,t}\right]}_{Ex}/{\left(\psi_{s}n_{s,t}\right)}^{2}+1}}\right),\ln\left(\frac{\hat{V}{\left[{\hat{n}}_{s,t}\right]}_{Ex}}{{\left(\psi_{s}n_{s,t}\right)}^{2}}+1\right)\right)$
	Use the external abundance estimate variances $\hat{V}{\left[{\hat{n}}_{s,t}\right]}_{Ex}$ but use the bias corrected latent abundance value $n_{s,t}$ to obtain the CV term in Equation (17).
3	${\hat{n}}_{s,t}∼\text{LogNormal}\left(\ln\left(\psi_{s}n_{s,t}\right)-\sigma_{O,s,In}^{2}/2,\sigma_{O,s,In}^{2}\right)$
	Do not use external information about the variances of ${\hat{n}}_{s,t}$, and instead internally estimate observation variance along with the other parameters and latent states of the SSM. In this case, it is not feasible to estimate both life stage and cohort specific values, but life stage‐specific (and possibly survey method specific if multiple surveys are used to monitor a single life stage) parameters may be estimable. The observation model equations are similar to Equations (9)–(12) but with a life stage *s* specific observation log‐variance parameter.

*Note*. The bias terms are included for generality with the understanding that $\psi_{s}=1$ if there is no bias term present in the life stage or survey specific observation model.

## 3 THEORETICAL IDENTIFIABILITY

We applied methods developed by Cole and McCrea (2016) to evaluate the theoretical identifiability of the parameters in the SSM described in Section 2 under various scenarios involving fixed or estimated observation error CV and the inclusion or exclusion of covariates in the state process model. An exhaustive summary vector based on (approximate) expected values and variances of the observations was derived assuming each state process had an intercept and either no covariates or one covariate. The derivative matrix, formed by taking partial derivatives of this vector with respect to the parameters of the model, was computed using Maple 17 (Maple, 2013) and code modified from the appendices of Cole and McCrea (2016). If the rank of the derivative matrix, *r*, equals the number of parameters, *p*, then all the model parameters are separately identifiable. If r<p, then *r* parameters or parameter combinations are identifiable, and Maple code from Cole and McCrea (2016) specifies which single parameters and combinations of parameters are identifiable.

One analysis examined the identifiability of parameters in the SSM defined by Equations (1)–(12) assuming a single covariate was used in the modeling of each of the four state processes. This model has 18 parameters: eight state process model parameters determining expected values (ζ_0_, ζ_1_, β_0_, β_1_, η_0_, η_1_, γ_0_, γ_1_), two observation bias parameters ($\psi_{J}$, $\psi_{SA}$), four process noise parameters ($\sigma_{P,R}^{2},\sigma_{P,PL}^{2}$, $\sigma_{P,J}^{2}$, $\sigma_{P,SA}^{2}$), and four observation variance parameters assuming that these parameters were time‐invariant ($\sigma_{O,PL}^{2}$, $\sigma_{O,J}^{2}$, $\sigma_{O,SA}^{2}$, $\sigma_{O,A}^{2}$). Crucially, the initial state component, $n_{A,0}$, was viewed as a known parameter, which seemed reasonable given the assumption of unbiased estimates of the abundance of adults in the year prior to the start of the time series. An exhaustive summary vector of length 20 (which proved to be of sufficient length) was constructed based on first‐order approximations of the expected values and variances of the observations. A subset of the vector including examples of the expectations and variances is

(18) $$E\left[{\hat{n}}_{PL,1}\right]=E\left[n_{PL,1}\right]\approx e^{\zeta_{0}+\zeta_{1}x_{R,1}}n_{A,0},$$

(19) $$\begin{array}{c} E\left[{\hat{n}}_{J,1}\right]=E\left[\psi_{J}n_{J,1}\right]\approx \psi_{J}\frac{e^{\beta_{0}+\beta_{1}x_{PL,1}}}{1+e^{\beta_{0}+\beta_{1}x_{PL,1}}}e^{\zeta_{0}+\zeta_{1}x_{R,1}}n_{A,0}, \end{array}$$

(20) $$\begin{array}{ccc} E[{\hat{n}}_{SA,1}] & = & E[\psi_{SA}n_{SA,1}]\\ & \approx & \psi_{SA}\frac{e^{\eta_{0}+\eta_{1}x_{J,1}}}{1+e^{\eta_{0}+\eta_{1}x_{J,1}}}\frac{e^{\beta_{0}+\beta_{1}x_{PL,1}}}{1+e^{\beta_{0}+\beta_{1}x_{PL,1}}}e^{\zeta_{0}+\zeta_{1}x_{R,1}}n_{A,0}, \end{array}$$

(21) $$\begin{array}{ccc} V[{\hat{n}}_{PL,1}] & = & \sigma_{O,PL}^{2}+V\left[\rho_{1}n_{A,0}\right]\approx \sigma_{O,PL}^{2}\\ & & +\;n_{A,0}^{2}{\left(e^{\zeta_{0}+\zeta_{1}x_{R,1}}\right)}^{2}\left(e^{\sigma_{P,R}^{2}}-1\right). \end{array}$$

The matrix of first derivatives of the exhaustive summary vector with respect to each of the parameters had a full rank of 18, thus all 18 parameters were identifiable. Further details on the exhaustive summary calculations for this SSM are provided in Web Appendix A.

A second set of analyses examined the effect of state process covariates on identifiability. This was largely motivated by the fact that observations for two of the life stages in the case study (Section 5) were biased. The potential for nonidentifiability can be seen in the case of a single life stage auto‐regressive process model fit with biased estimates. For example, given ${\hat{n}}_{1}$ ≈ $\psi n_{1}$ and *n*_1_ ≈ $e^{\zeta_{0}}n_{0}$, then $E[{\hat{n}}_{1}]$ ≈ $\psi e^{\zeta_{0}}n_{0}$ and difficulty in separating ψ and ζ_0_ is apparent. The case for multiple life stages, where unbiased estimates were available for some life stages, is more complicated and the effect of including covariates for different processes in the state process model was of interest. Focusing solely on estimability of mean parameters in the state and observation models, the effects on identifiability of a single covariate being present or absent in each of the four process models (2^4^ = 16 combinations) were examined. In the case where the derivative matrix is not full rank, additional Maple code developed by Cole and McCrea (2016) determines which parameters or combinations of parameters are identifiable.

The results of this second set of analyses are shown in Table 2. In the most limited setting where no covariates are used to model the process dynamics (case 1), there are six parameters, but only four parameter combinations can be estimated, and the only separately identifiable parameter is the intercept, ζ_0_, for the process model dynamics. The identifiability of ζ_0_ is readily seen given $n_{A,0}$ is assumed known and ${\hat{n}}_{PL,1}$ is unbiased for $n_{PL,1}$: $E[{\hat{n}}_{PL,1}]$ ≈ $e^{\zeta_{0}}n_{A,0}$. An example of an identifiable combination, also from case 1, is $\psi_{J}\left(e^{\beta_{0}}/\left(1+e^{\beta_{0}}\right)\right)$, which shows that the bias in estimates of juvenile abundance cannot be separated from post‐larval survival. When a covariate is used for a given process, the intercept and slope parameters for that process are always identifiable; for example, in case 3 a covariate for post‐larval survival is included and β_0_ and β_1_ are separately identifiable. When at least two survival process models have covariates (cases 9‐16) all the parameters are individually identifiable. Whether a bias parameter is identifiable is a function of the inclusion of covariates and which life stage abundance observation is biased. For example, parameter $\psi_{J}$ (but not $\psi_{SA}$) was identifiable when a covariate for the survival of post‐larvae to the juvenile stage was included (case 3), while $\psi_{SA}$ was identifiable when a covariate for the survival of sub‐adults to the adult stage was included (case 5). If covariates were available for all four processes, and observations for all four life stages were biased, that is, there were also $\psi_{PL}$ and $\psi_{A}$, say, all the parameters were identifiable (cases 17‐18).

**TABLE 2:** Summary of parameter identifiability results for state process mean parameters and observation bias parameters conditional on the inclusion or exclusion of covariates in a given set of state processes

Case	State processes with covariates	Parameters to be estimated (total)	Number of identifiable parameter combinations	Identifiable singleton parameters
1	None	{$\zeta_{0},\beta_{0},\eta_{0},\gamma_{0},\psi_{J},\psi_{SA}$} (6)	4	ζ_0_
2	ρ	{$\zeta_{0},\zeta_{1},\beta_{0},\eta_{0},\gamma_{0},\psi_{J},\psi_{SA}$} (7)	5	$\zeta_{0},\zeta_{1}$
3	$\phi_{PL}$	{$\zeta_{0},\beta_{0},\beta_{1},\eta_{0},\gamma_{0},\psi_{J},\psi_{SA}$} (7)	6	$\zeta_{0},\beta_{0},\beta_{1},\psi_{J}$
4	$\phi_{J}$	{$\zeta_{0},\beta_{0},\eta_{0},\eta_{1},\gamma_{0},\psi_{J},\psi_{SA}$} (7)	6	$\zeta_{0},\eta_{0},\eta_{1}$
5	$\phi_{SA}$	{$\zeta_{0},\beta_{0},\eta_{0},\gamma_{0},\gamma_{1},\psi_{J},\psi_{SA}$} (7)	6	$\zeta_{0},\gamma_{0},\gamma_{1},\psi_{SA}$
6	$\rho ,\phi_{PL}$	{$\zeta_{0},\zeta_{1},\beta_{0},\beta_{1},\eta_{0},\gamma_{0},\psi_{J},\psi_{SA}$} (8)	7	$\zeta_{0},\zeta_{1},\beta_{0},\beta_{1},\psi_{J}$
7	$\rho ,\phi_{J}$	{$\zeta_{0},\zeta_{1},\beta_{0},\eta_{0},\eta_{1},\gamma_{0},\psi_{J},\psi_{SA}$} (8)	7	$\zeta_{0},\zeta_{1},\eta_{0},\eta_{1}$
8	$\rho ,\phi_{SA}$	{$\zeta_{0},\zeta_{1},\beta_{0},\eta_{0},\gamma_{0},\gamma_{1},\psi_{J},\psi_{SA}$} (8)	7	$\zeta_{0},\zeta_{1},\gamma_{0},\gamma_{1},\psi_{SA}$
9	$\phi_{PL},\phi_{J}$	{$\zeta_{0},\beta_{0},\beta_{1},\eta_{0},\eta_{1},\gamma_{0},\psi_{J},\psi_{SA}$} (8)	8	All
10	$\phi_{PL},\phi_{SA}$	{$\zeta_{0},\beta_{0},\beta_{1},\eta_{0},\gamma_{0},\gamma_{1},\psi_{J},\psi_{SA}$} (8)	8	All
11	$\phi_{J},\phi_{SA}$	{$\zeta_{0},\beta_{0},\eta_{0},\eta_{1},\gamma_{0},\gamma_{1},\psi_{J},\psi_{SA}$} (8)	8	All
12	$\rho ,\phi_{PL},\phi_{J}$	{$\zeta_{0},\zeta_{1},\beta_{0},\beta_{1},\eta_{0},\eta_{1},\gamma_{0},\psi_{J},\psi_{SA}$} (9)	9	All
13	$\rho ,\phi_{PL},\phi_{SA}$	{$\zeta_{0},\zeta_{1},\beta_{0},\beta_{1},\eta_{0},\gamma_{0},\gamma_{1},\psi_{J},\psi_{SA}$} (9)	9	All
14	$\rho ,\phi_{J},\phi_{SA}$	{$\zeta_{0},\zeta_{1},\beta_{0},\eta_{0},\eta_{1},\gamma_{0},\gamma_{1},\psi_{J},\psi_{SA}$} (9)	9	All
15	$\phi_{PL},\phi_{J},\phi_{SA}$	{$\zeta_{0},\beta_{0},\beta_{1},\eta_{0},\eta_{1},\gamma_{0},\gamma_{1},\psi_{J},\psi_{SA}$} (9)	9	All
16	$\rho ,\phi_{PL},\phi_{J},\phi_{SA}$	{$\zeta_{0},\zeta_{1},\beta_{0},\beta_{1},\eta_{0},\eta_{1},\gamma_{0},\gamma_{1},\psi_{J},\psi_{SA}$} (10)	10	All
17	$\rho ,\phi_{PL},\phi_{J},\phi_{SA}$	{$\zeta_{0},\zeta_{1},\beta_{0},\beta_{1},\eta_{0},\eta_{1},\gamma_{0},\gamma_{1},\psi_{PL},\psi_{J},\psi_{SA},\psi_{A}$} (12)	12	All
18	$\rho ,\phi_{J},\phi_{SA}$	{$\zeta_{0},\zeta_{1},\beta_{0},\eta_{0},\eta_{1},\gamma_{0},\gamma_{1},\psi_{PL},\psi_{J},\psi_{SA},\psi_{A}$} (11)	11	All

*Note*. Bias parameter identifiability depends on covariate inclusion and where in the sequence of observations they occur. With juvenile and sub‐adult biases only (cases 1‐16), inclusion of a process covariate allows identifiability of the corresponding intercept and slope parameters (cases 2‐16) and all parameters are identifiable if at least two survival process models have covariates (cases 9‐16). With biases in all life stages and covariates in all processes, all parameters are identifiable (cases 17‐18).

These theoretical calculations are based on a frequentist model formulation, while in Sections 4 and 5 we apply Bayesian methods to fit the SSM. Nonidentifiability in a frequentist context implies nonidentifiability in a Bayesian context with uninformative priors, although it is possible for informative priors to help alleviate this issue (Cole and McCrea, 2016). Scenarios 1 and 2 in Table 1 assume a prior probability of 1 for the externally estimated observation variance values.

## 4 SIMULATION STUDY

### 4.1 Design

Although we identified conditions under which parameter identifiability is ensured theoretically, data‐specific features can result in practically nonidentifiable, or nearly redundant, parameters, sometimes identified by flat profile likelihoods and infinitely large confidence intervals in a frequentist context (Raue *et al*., 2009). We therefore used simulations to explore practical identifiability and to explore the potential benefits of fixing external estimates of $CV{\left[{\hat{n}}_{s,t}\right]}_{Ex}$ as in Scenario 1 (Table 1). Datasets were generated in R (R Core Team, 2019) according to Equations (1)‐(12) with 20 cohorts. Motivated by the case study (Section 5), the post‐larval and adult life stages were assumed to have no observation bias ($\psi_{PL}=\psi_{A}=1$) while the juvenile and sub‐adult life stages had observation biases less than one. Each dataset was used to fit two models, one with $CV[{\hat{n}}_{s,t}]$ fixed at externally derived estimates (Scenario 1) and one with $CV[{\hat{n}}_{s,t}]$ internally estimated as part of the model (Scenario 3). In the first model, the fixed values were assumed to be potentially imperfect according to the distribution $\hat{CV}{\left[{\hat{n}}_{s,t}\right]}_{Ex}∼\text{Unif}\left(\left(1-a\right)CV\left[{\hat{n}}_{s,t}\right],\left(1+a\right)CV\left[{\hat{n}}_{s,t}\right]\right)$, where $CV[{\hat{n}}_{s,t}]$ is the true value and a∈[0,1).

We ran 100 simulations each for a=0, in which case $\hat{CV}{\left[{\hat{n}}_{s,t}\right]}_{Ex}$ is equal to the true value $CV[{\hat{n}}_{s,t}]$, and for a=0.5, which represents a more realistic case where $\hat{CV}{\left[{\hat{n}}_{s,t}\right]}_{Ex}$ is estimated imperfectly. True values of $CV[{\hat{n}}_{s,t}]$ were generated from a Uniform(0.1, 1) distribution. The recruitment and three survival processes were each functions of single covariates. To incorporate the concept of model selection in the study along with parameter estimation, we included two potential covariates per process (the true covariate used to generate data and a second covariate) in the fitted models. The true values used to generate data and the prior distributions used for model fitting are described in Web Appendix B (Table B.1). Model fitting here and in Section 5 used Bayesian methods implemented within R (R Core Team, 2019) using JAGS v4.3.0 (Plummer, 2003; 2016; Su and Yajima, 2015). Model performance was evaluated by calculating marginal posterior summary statistics as well as relative bias, that is, (posteriorMean ‐ trueValue)/trueValue. Model convergence was assessed by examining trace plots for adequate mixing and calculating Gelman‐Rubin statistics.

### 4.2 Results

Posterior means of the vital rate coefficients, process variance, and observation bias parameters, averaged across simulations, were similar whether observation error was externally or internally estimated and whether the level of noise in the external estimates was low (a=0) or high (a=0.5) (Table 3). On average, observation bias parameters were well estimated, while recruitment process variance exhibited the highest level of relative bias (Table 3). Internally estimated observation CV was also generally biased high relative to the mean true observation CV for each life stage (Figure B.1). Plugging in external estimates of observation CV resulted in lower average posterior standard deviations and relative biases, as well as reduced diffusivity in joint posteriors (Figure B.2). Latent abundance posterior means were similarly well estimated regardless of how observation CV was handled, although plugging in external observation CV estimates can lead to smaller latent abundance standard deviations (Figure B.3). The findings presented here for the case with a=0.5 are qualitatively similar to those for the case with a=0.

**TABLE 3:** Summary of simulation study parameter estimates

		(a) Simulation with a=0.						(b) Simulation with a=0.5.					
	True	Mean		SD		Rel bias		Mean		SD		Rel bias	
Parameter	value	$M_{Ex}$	$M_{In}$	$M_{Ex}$	$M_{In}$	$M_{Ex}$	$M_{In}$	$M_{Ex}$	$M_{In}$	$M_{Ex}$	$M_{In}$	$M_{Ex}$	$M_{In}$
ζ_0_	1.00	1.02	1.07	0.12	0.18	0.02	0.07	1.03	1.08	0.11	0.18	0.03	0.08
ζ_1_	1.00	0.98	0.97	0.09	0.12	−0.02	−0.03	0.99	1.00	0.10	0.12	−0.01	0.00
ζ_2_	0.00	0.00	0.01	0.09	0.12	NA	NA	0.01	0.00	0.09	0.12	NA	NA
β_0_	1.30	1.33	1.28	0.60	0.67	0.02	−0.01	1.34	1.27	0.60	0.68	0.03	−0.02
β_1_	1.00	0.82	0.70	0.39	0.45	−0.18	−0.30	0.78	0.69	0.40	0.45	−0.22	−0.31
β_2_	0.00	0.01	0.00	0.36	0.43	NA	NA	0.00	−0.02	0.37	0.43	NA	NA
η_0_	1.30	1.34	1.32	0.60	0.67	0.03	0.01	1.32	1.28	0.60	0.66	0.02	−0.01
η_1_	1.00	0.80	0.68	0.40	0.46	−0.20	−0.32	0.82	0.71	0.40	0.45	−0.18	−0.29
η_2_	0.00	−0.01	0.02	0.36	0.43	NA	NA	−0.05	−0.04	0.36	0.43	NA	NA
γ_0_	1.30	1.40	1.37	0.59	0.66	0.08	0.05	1.46	1.43	0.60	0.67	0.12	0.10
γ_1_	1.00	0.85	0.76	0.40	0.46	−0.15	−0.24	0.75	0.66	0.42	0.47	−0.25	−0.34
γ_2_	0.00	−0.01	−0.05	0.37	0.43	NA	NA	0.02	−0.01	0.39	0.45	NA	NA
$\sigma_{P,R}$	0.05	0.11	0.15	0.09	0.12	1.27	1.93	0.15	0.15	0.10	0.11	2.04	2.01
$\sigma_{P,PL}$	0.50	0.48	0.53	0.36	0.43	−0.04	0.06	0.58	0.56	0.41	0.44	0.16	0.12
$\sigma_{P,J}$	0.50	0.52	0.58	0.38	0.45	0.03	0.16	0.53	0.55	0.39	0.43	0.07	0.10
$\sigma_{P,SA}$	0.50	0.51	0.55	0.38	0.44	0.01	0.10	0.59	0.61	0.42	0.46	0.18	0.21
$\psi_{J}$	0.50	0.52	0.55	0.08	0.15	0.04	0.10	0.51	0.53	0.08	0.14	0.03	0.07
$\psi_{SA}$	0.20	0.21	0.23	0.03	0.06	0.05	0.13	0.21	0.23	0.03	0.06	0.05	0.15

*Note*. Average posterior mean (Mean), posterior standard deviation (SD), and relative bias (Rel Bias) were calculated across simulations for the cases with a=0 and a=0.5. Externally estimated CV equals true CV when a=0, and randomly varies from 50% to 150% of the true CV when a=0.5. $M_{Ex}$ and $M_{In}$ represent the models with externally and internally estimated observation error, respectively. Relative biases for the coefficients ψ_2_, β_2_, η_2_, and γ_2_ are undefined because the true values are zero.

## 5 CASE STUDY

We used the model described in Section 2 to quantify the relative importance of salient factors posited to determine the population dynamics of delta smelt (*Hypomesus transpacificus*). Delta smelt are a small, nearly annual pelagic fish endemic to the interior “Delta” portion of the San Francisco Estuary (Moyle *et al*., 1992; 2016). Spawning typically takes place during the winter and spring, with offspring maturing through a number of life stages to eventually become spawning adults by winter of the next calendar year.

Abundance indices and coefficients of variation developed in Polansky *et al*. (2019) were used as observations on the abundances of each life stage starting with the 1994 birth cohort adult abundance index and including the subsequent 21 cohorts. Based on the abundance index construction work in Polansky *et al*. (2019), we assumed post‐larval indices, and adult indices for t>6, were unbiased relative to an overall unknown scaling factor, that is, $\psi_{PL}=1$ and $\psi_{A,t}=1$ for t>6. Juvenile and sub‐adult observation biases, $\psi_{J}$ and $\psi_{SA}$, respectively, were estimated. Because the sampling method used to collect data about adult abundances prior to 2002 was the same as that used for sub‐adult data collection, and indices include length based corrections, we set $\psi_{A,t}=\psi_{SA}$ for t≤6. Covariate data used to model recruitment and survival consisted of a collection of abiotic habitat condition metrics, abundance indices of bottom up and top down trophic drivers, and competitors summarized in Web Appendix C Table C.1.

The steps of assembling covariate sets to model each vital rate and the complete set of parameter posterior results are described in detail in Web Appendix C. Model validation was done in several ways, including graphical posterior checks of response residuals (Gelman and Shalizi, 2013), and q‐q plots of one step ahead forecast residuals (Smith, 1985).

Two “global” models with the same sets of covariates for each vital rate were considered: one that used external estimates of observation error CV (Scenario 1), and one that used internally estimated observation error CV (Scenario 3). Posterior summaries are provided in Table C.4. Process noise variance estimates in the model fit using Scenario 1 were higher than those estimated under Scenario 3, while the internally estimated observation coefficients of variation were generally larger than the externally derived ones (Figure C.3). As predicted by the simulation study, Scenario 1 posterior standard deviations of latent abundances and observation bias were smaller compared to those estimated under Scenario 3 (Table C.4 and Figure C.4). Posterior distributions of the bias parameters in both cases showed them to be considerably smaller than one. The model from Scenario 2 was not applied because it is similar to the model from Scenario 1, and the external estimates of abundance variance, $\hat{V}{\left[{\hat{n}}_{s,t}\right]}_{Ex}$, were expected to be as informative as the external estimates of abundance CV, $\hat{CV}{\left[{\hat{n}}_{s,t}\right]}_{Ex}$.

We found a number of covariates with more support than others for each vital rate (Table C.4). Here we present a subset of these results to illustrate the most important vital rate predictions. Using the results from the global model fit with external estimates of observation error CV plugged in, and selecting (somewhat arbitrarily) a 0.80 value as the lower limit for which evidence, the posterior distribution probability that the coefficient is above (below) zero when the expected effect of a covariate is positive (negative), is considered substantial enough to report on here, the following relationships were observed: (a) recruitment was most influenced by temperature, the approximate location of the 2‐ppt isohaline during the previous fall, and adult food (note also the export‐inflow ratio had high evidence of support based on the models summarized in Table C.2); (b) post‐larval survival by outflow and turbidity; (c) juvenile survival by turbidity (Secchi depth) and temperature; and (d) sub‐adult survival by turbidity in the south Delta (south Secchi depth), a spatially localized hydrodynamics flow measure in the Old and Middle River corridor (OMR), and adult striped bass (*Morone saxatilis*). Of the predator/competitor indices considered here, only the effect of summer inland silverside (*Menidia beryllina*) abundance on post‐larval survival, and juvenile and adult striped bass on sub‐adult survival, had biologically plausible negative estimated effects.

**FIGURE 1:**
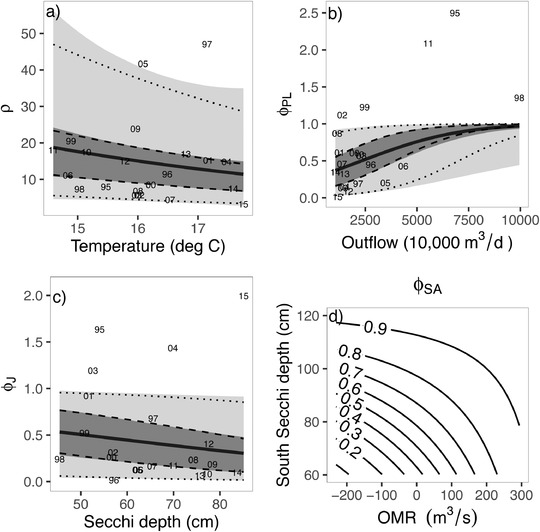
Predicted vital rates for the case study example model. In all panels the solid curved lines show expected values. In panels (a)‐(c), dark and light gray shadings show the 100(1−α)% central credible intervals for α=0.5 and α=0.05, respectively, and include posterior parameter estimate uncertainty. The dashed and dotted lines show the 50% and 95%, respectively, central credible intervals using the mean values of the posterior. Ratios of abundance indices are shown with the last two digits of the cohort year, adjusted by the inverse of the posterior means of observation error bias when relevant. Covariate units are mean daily values over the time interval of each vital rate [Correction added on 8 October 2020, after first online publication: Author corrected labels and units]

To illustrate vital rate predictions, a model including only the covariates with the highest posterior evidence for each vital rate was constructed. Also included was an interaction between the two covariates that most impact sub‐adult survival, both of which had near one evidence of support. There is little difference between the distribution of vital rate predictions when parameter estimate uncertainty is not included, and the interquartile prediction range is considerably more bounded than the 95% prediction interval (Figure 1). Increases in sub‐adult survival with decreases in turbidity (increases in south Secchi) become more pronounced as OMR decreases (Figure 1).

## 6 DISCUSSION

Identifiability issues for SSMs fit to ecological datasets have primarily concentrated on normal dynamic linear models (de Valpine and Hilborn (2005) and de Valpine and Rosenheim (2008) provide some exceptions), with specific focus on accurately distinguishing the magnitudes of process and observation variances (Dennis *et al*., 2006, 2010; Knape *et al*., 2011; Auger‐Méthé *et al*., 2016), and difficulties in accurately assessing density dependence in SSMs for population dynamics (Freckleton *et al*., 2006; Knape, 2008). The effects of covariates and the effects of external estimates of observation variance on identifiability, particularly for nonlinear and non‐Gaussian SSMs, have not received as much attention.

For the nonlinear non‐Gaussian SSMs examined here, the utility of covariates for enabling identifiability, particularly in the case of biased observations, was clear. Without covariates, but with bias in some of the observations, nonidentifiability occurs. In some cases, one can use method of moments to determine identifiability by setting the observations to their expected values and solving the resulting system of equations. However, the algebra involved in this approach can be extremely difficult and determining what parameter combinations are identifiable can also be challenging. The recently developed methods of Cole and McCrea (2016) are attractive and elegant tools for analytically assessing parameter identifiability for SSMs in a far less algebraically tedious manner, indicating both how many parameter combinations are identifiable as well as what they are. These methods deserve routine application both to guide SSM formulation as well as for after‐the‐fact assessment. A cautionary note, however, is that theoretical identifiability does not rule out practical nonidentifiability for a given dataset (Raue *et al*., 2009). Simulating datasets from a hypothesized SSM and then examining the ability to estimate the known parameters can be a helpful exercise for identifying practical estimation difficulties and data specific modeling challenges. We note that data cloning (Lele *et al*., 2010), while not applied here, is an alternative method of determining estimability of model parameters and functions of parameters that relies only on the observed data.

Based on the simulation study, the improvement in inference when observation error CV was externally estimated (Scenario 1) compared to when it was internally estimated (Scenario 3) was relatively minor, given the presence of modeled covariate effects. The primary advantage of using external estimates was an increase in precision, particularly for the observation bias and state estimates. As expected, these gains decrease when the external estimates of observation variance are themselves measured with error. Case study results based on these two scenarios mostly mirror the simulation study findings, particularly with respect to inference about latent states and observation bias parameters. However, an important difference is that when observation error CV was estimated internally, estimation difficulties appeared to be greatly exacerbated in general, with complex joint posterior distributions containing multiple modes and ridges, particularly in the process variance and intercept parameter dimensions. One consequence was that process variance estimates were unrealistically low. The exact aspect of the case study dataset responsible for the low process noise estimates is not clear. A longer time series may help separate process variance from observation variance. However, process variance estimates were not unrealistically low in the simulation study when only 20 cohorts were used, suggesting that unidentified sources of bias or uncertainty (or both) remain in the case study when observation error CV is estimated.

A practical concern when modeling empirical data is that relationships between covariates and response variables may appear weaker than they are in reality because of noise in the covariate data, also known as error‐in‐variables (Carroll *et al*., 2006), or because the covariate data were summarized in a nonoptimal way (Ferguson *et al*., 2017). Exploratory simulations indicated that covariate noise affects estimated vital rates in the SSM presented here, making this an aspect of the model that requires further development. An estimation issue that seems particularly challenging given the model framework considered here is that as covariate effect size diminishes, nonidentifiability issues can emerge. Including covariates does not guarantee that straightforward application of computational methods will be sufficient for estimation.

The delta smelt population modeling presented here integrated data from more surveys than has been done previously. Although the sequential life stage model required addressing relative bias in abundance estimates, this model framework allowed new insights about drivers of population abundances. For example, we found flow related impacts on summer survival and lagged fall flow effects on recruitment, whereas prior analyses by Feyrer *et al*. (2011) were unable to precisely identify where in the life cycle the flow effects on population dynamics occur. Extending the findings by Grimaldo *et al*. (2009), who found that OMR and turbidity predict an index of south Delta mortality, we found these spatially localized predictor variables interact and predict population wide sub‐adult survival. A number of predator/competitor relationships with delta smelt vital rates were biologically implausible, suggesting that some cohabitant species are more influenced in the contemporary Delta by shared habitat conditions than inter‐specific interactions.

Auger‐Méthé *et al*. (2016) remarked that SSMs are “becoming the favoured statistical framework [in ecology] for modelling animal movement and population dynamics.” The state‐process equations describing realistic population models are often nonlinear, non‐Gaussian, and link multiple life stages. Because SSMs allow integration of multiple datasets, they are an important tool for advancing population modeling in practice. Here we focused on a particular kind of population in which different age classes are not concurrently observed. More general populations with multiple age classes observed at the same time such as those as described by matrix models can encompass a wide variety of life history strategies (Caswell, 2001), and the number of possible configurations where biases and covariates appear can be quite numerous in general. Mapping out the theoretical and practical identifiability requirements for a given SSM remains an ongoing topic of research.

## Supporting information

Web Appendices, Tables, and Figures referenced in Sections 3, 4, and 5, Maple code to reproduce the theoretical identifiability assessments, and JAGS code to reproduce the results of the case study are available with this paper at the Biometrics website on Wiley Online Library.

## Data Availability

The data that support the findings in this paper are available in the Supporting Information of this article.
