# Supplementary material for: Improving inference for nonlinear state‐space models of animal population dynamics given biased sequential life stage data
Source: Biometrics. 2020 Apr 25;77(1):352–61. doi: 10.1111/biom.13267 (PMC7984174; doi:10.1111/biom.13267)
Supplement: Supplementary file 1 — Web Appendices, Tables, and Figures referenced in Sections 3, 4, and 5, Maple code to reproduce the theoretical identifiability assessments, and JAGS code to reproduce the results of the case study are available with this paper at the Biometrics website on Wiley Online Library. [file BIOM-77-352-s001.zip › biom13267-sup-0001-SuppMat.pdf]

Supporting Information for "Improving inference for nonlinear state-space models of animal population dynamics given biased sequential life stage data"
by
L. Polansky, K. B. Newman, and L. Mitchell

Web Appendix A
Analytical derivation of parameter identifiability

This section applies the methods of Cole and McCrea (2016), and in particular following their Web Appendix A.4, to show that the mean and variance parameters of the state-space model described in Section 2 are estimable if there is at least one covariate for each vital rate model. For simplicity, only one covariate per vital rate is assumed. Furthermore, while external and time-varying estimates of observation variance are available for the case study, we considered the situation where such estimates were not available but the observation variances were life stage specific but otherwise time invariant. The resulting parameter vector has 18 components: $\boldsymbol{\theta}$=(ζ_0, ζ_1, β_0, β_1, η_0, η_1, γ_0, γ_1, ψ_J, ψ_{SA}, $\sigma^2_{P,R}$, $\sigma^2_{P,PL}$, $\sigma^2_{P,J}$, $\sigma^2_{P,SA}$, $\sigma^2_{O,PL}$, $\sigma^2_{O,J}$, $\sigma^2_{O,SA}$, $\sigma^2_{O,A}$).

In the following we partition the calculation of the exhaustive summary into three pieces, one for the mean parameters of the state and observation models (namely the covariate coefficients and the observation bias parameters), another for the variance parameters of the state model, and another for the variance parameters of the observation model. We note that the initial analysis with `Maple` proceeded sequentially, starting with the first piece, then adding the second piece, and finally adding the third piece. In each case, all parameters were separately identifiable at each stage (10, then 14, and then 18 parameters).

Exhaustive summaries of covariate coefficients and observation biases. For simplicity we assumed just a single covariate per process, as the exhaustive summary vector would simply need to be extended in the case of more covariates, and assume that $n_{A,0}$ is known. Given an unbiased estimate of $n_{A,0}$ (namely, 1994 adults), $n_{A,0}$ is identifiable. That leaves 10 unknown parameters. Thus at least 10 components to the exhaustive summary are needed. However, based on preliminary analyses with `Maple`, the "first" 12 observations were needed.

The lognormal bias corrections in the observations simplify the expected values of the observations (compared to non-bias corrections); e.g. $E[\widehat{n}_{PL,1}] = E[E[\widehat{n}_{PL,1}|n_{PL,1}]] = E[n_{PL,1}]$.

$$
\begin{aligned}
E[\widehat{n}_{PL,1}] &= E[n_{PL,1}] \approx e^{\zeta_0+\zeta_1 x_{R,1}} n_{A,0} \\
E[\widehat{n}_{J,1}] &= E[\psi_J n_{J,1}] \approx \psi_J \frac{e^{\beta_0+\beta_1 x_{PL,1}}}{1+e^{\beta_0+\beta_1 x_{PL,1}}} e^{\zeta_0+\zeta_1 x_{R,1}} n_{A,0} \\
E[\widehat{n}_{SA,1}] &= E[\psi_{SA} n_{SA,1}] \approx \psi_{SA} \frac{e^{\eta_0+\eta_1 x_{J,1}}}{1+e^{\eta_0+\eta_1 x_{J,1}}} \frac{e^{\beta_0+\beta_1 x_{PL,1}}}{1+e^{\beta_0+\beta_1 x_{PL,1}}} e^{\zeta_0+\zeta_1 x_{R,1}} n_{A,0} \\
E[\widehat{n}_{A,1}] &= E[n_{A,1}] \approx \frac{e^{\gamma_0+\gamma_1 x_{SA,1}}}{1+e^{\gamma_0+\gamma_1 x_{SA,1}}} \frac{e^{\eta_0+\eta_1 x_{J,1}}}{1+e^{\eta_0+\eta_1 x_{J,1}}} \frac{e^{\beta_0+\beta_1 x_{PL,1}}}{1+e^{\beta_0+\beta_1 x_{PL,1}}} e^{\zeta_0+\zeta_1 x_{R,1}} n_{A,0} \\
E[\widehat{n}_{PL,2}] &= E[n_{A,2}] \approx e^{\zeta_0+\zeta_1 x_{R,2}} \frac{e^{\gamma_0+\gamma_1 x_{SA,1}}}{1+e^{\gamma_0+\gamma_1 x_{SA,1}}} \frac{e^{\eta_0+\eta_1 x_{J,1}}}{1+e^{\eta_0+\eta_1 x_{J,1}}} \frac{e^{\beta_0+\beta_1 x_{PL,1}}}{1+e^{\beta_0+\beta_1 x_{PL,1}}} e^{\zeta_0+\zeta_1 x_{R,1}} n_{A,0} \\
&\vdots \\
E[\widehat{n}_{A,3}] &= \ldots
\end{aligned}
$$

where the expectations are approximated by the deterministic version of the model.

Letting $z_t = e^{\zeta_0+\zeta_1 x_{R,t}}$, $b_t = e^{\beta_0+\beta_1 x_{PL,t}}/(1+e^{\beta_0+\beta_1 x_{PL,t}})$, $e_t = e^{\eta_0+\eta_1 x_{J,t}}/(1+e^{\eta_0+\eta_1 x_{J,t}})$, and $g_t =$

$e^{\gamma_0+\gamma_1 x_{SA,t}}/(1+e^{\gamma_0+\gamma_1 x_{SA,t}})$, a length 12 exhaustive summary can be written as follows:

$$
\begin{aligned}
E[\widehat{n}_{PL,1}] &\approx z_1 n_{A,0} && \text{(A.1)}\\
E[\widehat{n}_{J,1}] &\approx \psi_J b_1 z_1 n_{A,0} && \text{(A.2)}\\
E[\widehat{n}_{SA,1}] &\approx \psi_{SA} e_1 b_1 z_1 n_{A,0} && \text{(A.3)}\\
E[\widehat{n}_{A,1}] &\approx g_1 e_1 b_1 z_1 n_{A,0} && \text{(A.4)}\\
E[\widehat{n}_{PL,2}] &\approx z_2 g_1 e_1 b_1 z_1 n_{A,0} && \text{(A.5)}\\
E[\widehat{n}_{J,2}] &\approx \psi_J b_2 z_2 g_1 e_1 b_1 z_1 n_{A,0} && \text{(A.6)}\\
E[\widehat{n}_{SA,2}] &\approx \psi_{SA} e_2 b_2 z_2 g_1 e_1 b_1 z_1 n_{A,0} && \text{(A.7)}\\
E[\widehat{n}_{A,2}] &\approx g_2 e_2 b_2 z_2 g_1 e_1 b_1 z_1 n_{A,0} && \text{(A.8)}\\
E[\widehat{n}_{PL,3}] &\approx z_3 g_2 e_2 b_2 z_2 g_1 e_1 b_1 z_1 n_{A,0} && \text{(A.9)}\\
E[\widehat{n}_{J,3}] &\approx \psi_J b_3 z_3 g_2 e_2 b_2 z_2 g_1 e_1 b_1 z_1 n_{A,0} && \text{(A.10)}\\
E[\widehat{n}_{SA,3}] &\approx \psi_{SA} e_3 b_3 z_3 g_2 e_2 b_2 z_2 g_1 e_1 b_1 z_1 n_{A,0} && \text{(A.11)}\\
E[\widehat{n}_{A,3}] &\approx g_3 e_3 b_3 z_3 g_2 e_2 b_2 z_2 g_1 e_1 b_1 z_1 n_{A,0} && \text{(A.12)}
\end{aligned}
$$

Exhaustive summary for state process variance parameters. Following Cole and McCrea (2016; based on their Web Appendix A.4), to determine the identifiability of the variance parameters the exhaustive summary is expanded to include *unconditional* variances of observations.

Two general probability results that are used repeatedly to approximate the unconditional variances of the observations are:

1. For two independent random variables X and Y:

$$V[XY] = V[X]E[Y]^2 + E[X]^2V[Y] + V[X]V[Y]$$

2. $V[Y] = E[V[Y|X]] + V[E[Y|X]]$. In the application here Y is the observation and X is the underlying state or the product of an observation bias parameter (ψ_J or ψ_{SA}) and the state. For example,

$$
\begin{aligned}
V[\widehat{n}_{PL,1}] &= E[V[\widehat{n}_{PL,1}|n_{PL,1}]] + V[E[\widehat{n}_{PL,1}|n_{PL,1}]]\\
&= \sigma^2_{O,PL,1} + V[n_{PL,1}]
\end{aligned}
$$

Focusing on exhaustive summaries for the four process variances, the variances of the first four observations are considered:

$$
\begin{aligned}
V[\widehat{n}_{PL,1}] &= \sigma^2_{O,PL,1} + V[n_{PL,1}] = \sigma^2_{O,PL,1} + V[\rho_1 n_{A,0}] = \sigma^2_{O,PL,1} + n^2_{A,0}V[\rho_1] && \text{(A.13)}\\
V[\widehat{n}_{J,1}] &= \sigma^2_{O,J,1} + V[\psi_J n_{J,1}] = \sigma^2_{O,J,1} + (\psi_J n_{A,0})^2 V[\phi_{PL,1} * \rho_1] && \text{(A.14)}\\
V[\widehat{n}_{SA,1}] &= \sigma^2_{O,SA,1} + V[\psi_{SA} n_{SA,1}] = \sigma^2_{O,SA,1} + (\psi_{SA} n_{A,0})^2 V[\phi_{J,1} * \phi_{PL,1} * \rho_1] && \text{(A.15)}\\
V[\widehat{n}_{A,1}] &= \sigma^2_{O,A,1} + V[n_{A,1}] = \sigma^2_{O,A,1} + n^2_{A,0} V[\phi_{SA,1} * \phi_{J,1} * \phi_{PL,1} * \rho_1] && \text{(A.16)}
\end{aligned}
$$

where, letting

$$
\begin{aligned}
s_1 &= e^{\sigma^2_{P,R}} - 1\\
s_{2,1} &= \frac{\sigma^2_{P,PL}}{(1+e^{\beta_0+\beta_1 x_{PL,1}})^2}\\
s_{3,1} &= \frac{\sigma^2_{P,J}}{(1+e^{\eta_0+\eta_1 x_{J,1}})^2}\\
s_{4,1} &= \frac{\sigma^2_{P,SA}}{(1+e^{\gamma_0+\gamma_1 x_{SA,1}})^2}
\end{aligned}
$$

$$
\begin{aligned}
V[\rho_1] &= z_1^2 s_1 \\
V[\phi_{PL,1} * \rho_1] &\approx b_1^2 z_1^2 (s_{2,1} + s_1 + s_{2,1} s_1) \\
V[\phi_{J,1} * \phi_{PL,1} * \rho_1] &\approx e_1^2 b_1^2 z_1^2 \left[s_{3,1}(1 + s_{2,1} + s_1 + s_{2,1} s_1) + (s_{2,1} + s_1 + s_{2,1} s_1) \right] \\
V[\phi_{SA,1} * \phi_{J,1} * \phi_{PL,1} * \rho_1] &\approx g_1^2 e_1^2 b_1^2 z_1^2 \left[s_{4,1} + (1 + s_{4,1})\{ s_{3,1}(1 + s_{2,1} + s_1 + s_{2,1} s_1) + (s_{2,1} + s_1 + s_{2,1} s_1) \} \right]
\end{aligned}
$$

where the variance for ρ_1, which is based on a lognormal random variable, is exact but the remaining variances are approximations of a logit-normal variance calculated using the delta method.

Exhaustive summary for observation variance parameters. We assume that observation variances are life stage, but not time, specific, and the exhaustive summary is extended with four more components. This is done by calculating the variances of the next four observations, namely, $\widehat{n}_{PL,2}$, $\widehat{n}_{J,2}$, $\widehat{n}_{SA,2}$, and $\widehat{n}_{A,2}$:

$$V[\widehat{n}_{PL,2}] = \sigma^2_{O,PL} + V[n_{PL,2}] = \sigma^2_{O,PL} + n^2_{A,0} V[\rho_2 \ \phi_{SA,1} \ \phi_{J,1} \ \phi_{PL,1} \ \rho_1] \quad \text{(A.17)}$$

$$V[\widehat{n}_{J,2}] = \sigma^2_{O,J} + V[\psi_J n_{J,2}] = \sigma^2_{O,J} + (\psi_J n_{A,0})^2 V[\phi_{PL,2} \ \rho_2 \ \phi_{SA,1} \ \phi_{J,1} \ \phi_{PL,1} \ \rho_1] \quad \text{(A.18)}$$

$$V[\widehat{n}_{SA,2}] = \sigma^2_{O,SA} + V[\psi_{SA} n_{SA,2}] = \sigma^2_{O,SA} + (\psi_{SA} n_{A,0})^2 V[\phi_{J,2} \ \phi_{PL,2} \ \rho_2 \ \phi_{SA,1} \ \phi_{J,1} \ \phi_{PL,1} \ \rho_1] \quad \text{(A.19)}$$

$$V[\widehat{n}_{A,2}] = \sigma^2_{O,A} + V[n_{A,2}] = \sigma^2_{O,A} + n^2_{A,0} V[\phi_{SA,2} \ \phi_{J,2} \ \phi_{PL,2} \ \rho_2 \phi_{SA,1} \ \phi_{J,1} \ \phi_{PL,1} \ \rho_1] \quad \text{(A.20)}$$

where

$$
\begin{aligned}
V[\rho_2 \ \phi_{SA,1} \ \phi_{J,1} \ \phi_{PL,1} \ \rho_1] &\approx V[\rho_2](g_1^2 e_1^2 b_1^2 z_1^2) + (V[\rho_2] + E[\rho_2]^2) V[\phi_{SA,1} \phi_{J,1} \phi_{PL,1} \rho_1] \\
&= z_2^2 s_1 (g_1^2 e_1^2 b_1^2 z_1^2) + (z_2^2 s_1 + z_2^2) V[\phi_{SA,1} \phi_{J,1} \phi_{PL,1} \rho_1] \\
V[\phi_{PL,2} \ \rho_2 \ \phi_{SA,1} \ \phi_{J,1} \ \phi_{PL,1} \ \rho_1] &\approx b_2^2 s_{2,2} (z_2^2 g_1^2 e_1^2 b_1^2 z_1^2) + (b_2^2 s_{2,2} + b_2^2) V[\rho_2 \ \phi_{SA,1} \ \phi_{J,1} \ \phi_{PL,1} \ \rho_1] \\
V[\phi_{J,2} \ \phi_{PL,2} \ \rho_2 \ \phi_{SA,1} \ \phi_{J,1} \ \phi_{PL,1} \ \rho_1] &\approx e_2^2 s_{3,2} (b_2^2 z_2^2 g_1^2 e_1^2 b_1^2 z_1^2) + (e_2^2 s_{3,2} + e_2^2) V[\phi_{PL,2} \ \rho_2 \ \phi_{SA,1} \ \phi_{J,1} \ \phi_{PL,1} \ \rho_1] \\
V[\phi_{SA,2} \ \phi_{J,2} \ \phi_{PL,2} \ \rho_2 \phi_{SA,1} \ \phi_{J,1} \ \phi_{PL,1} \ \rho_1] &\approx g_2^2 s_{4,2} (e_2^2 b_2^2 z_2^2 g_1^2 e_1^2 b_1^2 z_1^2) + (g_2^2 s_{4,2} + g_2^2) V[\phi_{J,2} \ \phi_{PL,1} \ \phi_{PL,2} \ \rho_2 \ \phi_{SA,1} \ \phi_{J,1} \ \phi_{PL,1} \ \rho_1]
\end{aligned}
$$

where $s_{2,2}$, $s_{3,2}$, and $s_{4,2}$ are analogous to $s_{2,1}$, $s_{3,1}$, and $s_{4,1}$ but using the covariates for time $t = 2$.

Results. The exhaustive summary vector for the 18 parameters consisted of the expressions from the three blocks of equations A.1-A.12, A.13-A.16, and A.17-A.20. The derivative matrix, D, of dimension 18 by 20, which was symbolically calculated using `Maple`, had rank 18, indicating that all 18 parameters are identifiable.

References

Cole, D. J. and McCrea, R. S. (2016). Parameter redundancy in discrete state-space and integrated models. *Biometrical Journal* **58,** 1071–1090.

Web Appendix B

Simulation study details

Table B.1: Simulation input values and prior distributions. Uniform distributions are written in terms of the minimum and maximum values, normal distributions are written in terms of the means and standard deviations, and exponential distributions are written in terms of the rate parameters. Prior distributions for the survival coefficients (β_i, η_i, γ_i, $i = 0, 1, 2$) were chosen such that the induced priors on the survival probabilities were Uniform$(0, 1)$ following Newman (2003).

Description	Parameter	True Value	Prior Distribution
Initial abundance	$n_{A,0}$	100,000	Uniform$(10000, 2000000)$
Recruitment	ζ_0	1	Normal$(0, 1)$
	ζ_1	1	Normal$(0, 1)$
	ζ_2	0	Normal$(0, 1)$
	$\sigma_{P,R}$	0.05	Exponential(5)
Survival			
ϕ_{PL}	β_0	1.3	Normal$(0, \sqrt{\pi^2/9})$
	β_1	1	Normal$(0, \sqrt{\pi^2/9})$
	β_2	0	Normal$(0, \sqrt{\pi^2/9})$
	$\sigma_{P,PL}$	0.5	Exponential(0.75)
ϕ_J	η_0	1.3	Normal$(0, \sqrt{\pi^2/9})$
	η_1	1	Normal$(0, \sqrt{\pi^2/9})$
	η_2	0	Normal$(0, \sqrt{\pi^2/9})$
	$\sigma_{P,J}$	0.5	Exponential(0.75)
ϕ_{SA}	γ_0	1.3	Normal$(0, \sqrt{\pi^2/9})$
	γ_1	1	Normal$(0, \sqrt{\pi^2/9})$
	γ_2	0	Normal$(0, \sqrt{\pi^2/9})$
	$\sigma_{P,SA}$	0.5	Exponential(0.75)
Observation measurement bias			
	ψ_J	0.5	Exponential(1)
	ψ_{SA}	0.2	Exponential(1)
Observation measurement coefficient of variation	$\widehat{CV}[\widehat{n}_{s,t}]_{Ex}$	Uniform$(0.1, 1)$	Uniform$(0.0001, 7)$

References

Newman, K. B. (2003). Modelling paired release-recovery data in the presence of survival and capture heterogeneity with application to marked juvenile salmon. *Statistical Modelling* **3,** 157–177.

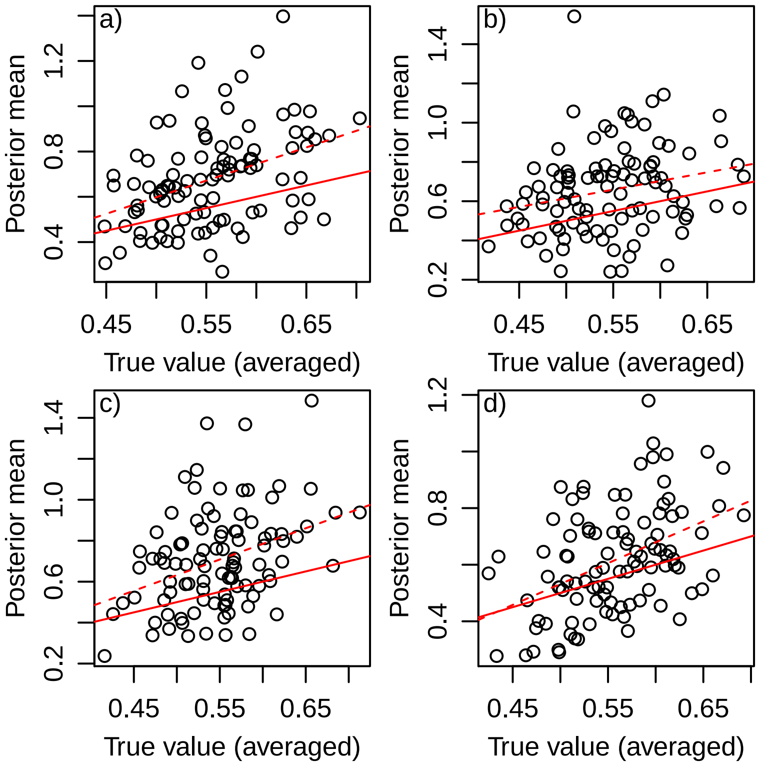

Figure B.1: Posterior mean of the observation error CV plotted against the true CV (averaged over cohort years) for a) post-larval, b) juvenile, c) sub-adult, and d) adult life stages based on the simulation study with $a = 0.5$. One-to-one lines (solid) and fitted linear regression lines (dashed) are shown on each panel for reference.

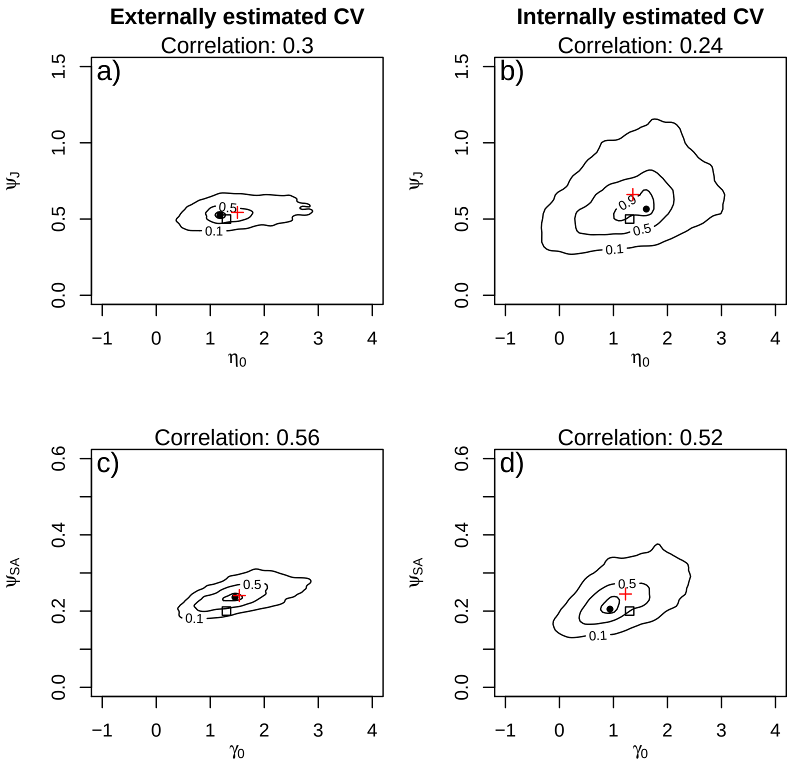

Figure B.2: Joint posterior density plots of the observation bias parameter and survival intercept for juvenile (panels a and b) and sub-adult (panels c and d) survival sub-processes based on a single simulation with $a = 0.5$. The left and right columns correspond to externally and internally estimated observation error CV. Joint density plots are scaled to lie between 0 and 1, levels are drawn at the 0.9, 0.5, and 0.1 quantiles, boxes reflect true parameter values, dots are at the marginal maximums, and crosses are at the marginal means.

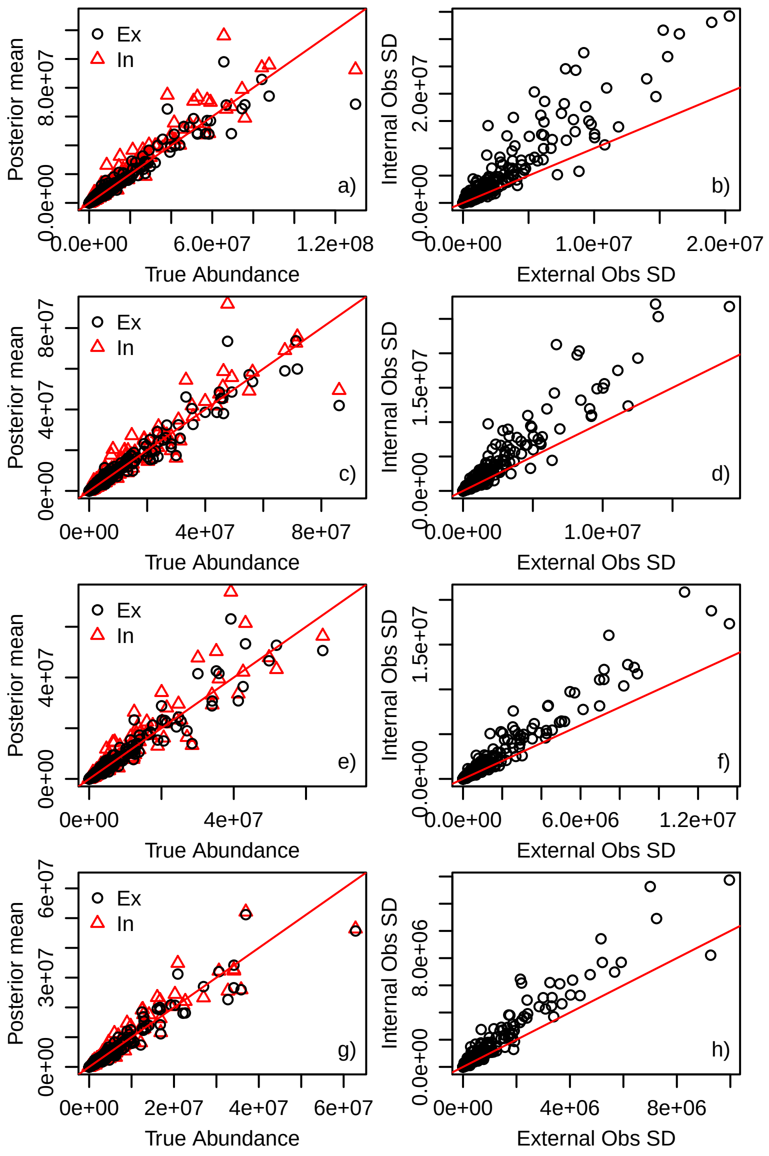

Figure B.3: Latent abundance posterior means (left column) and standard deviations (right column) for post-larval (panels a and b), juvenile (panels c and d), sub-adult (panels e and f), and adult (panels g and h) life stages based on the simulation study with $a = 0.5$. Left column shows posterior mean vs. true abundance for models with externally (Ex) and internally (In) estimated observation error CV. Right column shows posterior standard deviations for abundances (post-larval, juvenile, sub-adult, and adult) from the model with internal observation error CV estimates (Internal Obs CV) against standard deviations from the model with external estimates (External Obs CV). A one-to-one line is shown in each panel for reference.

Web Appendix C
Case study details

Covariate data were obtained from a variety of California State and U.S. Federal government agencies. Data collected from areas of the Delta most likely to coincide with delta smelt habitat were aggregated into multi-month summaries using means or medians and then standardized prior to model fitting. Table C.1 describes the types of data considered. For exploratory and management purposes, more predictor variables for any given vital rate were constructed than could be simultaneously included in a single "global" model that includes multiple covariates per vital rate because of collinearity, e.g. spring inflow, outflow, and OMR, or summer flows or X2 and NJACM.

Prior to fitting global models, we fit a series of relatively simple models that used exactly one predictor variable for each vital rate, each of which was of the same type, e.g. an all inflow model used spring, summer, fall and winter measures of inflow to predict each of the corresponding vital rates. This was helpful for identifying the strength of a relationship for each covariate alone, by examining the coefficient values and process noise variance estimates, when including other ones measuring similar features of the ecosystem would be difficult because of collinearity. The results also give a preliminary indication about whether any vital rates show a response in the direction opposite that expected and thus warrant further consideration of inclusion based on biological sensibility.

Posterior summaries of process parameters and graphical descriptions of vital rate predictions were used to assess these relatively simple models. Vital rate prediction intervals that included posterior uncertainty were constructed by first sampling a vector of parameters from the joint posterior distribution of the fitted model, and then simulating a realized vital rate given this sample, repeated 10,000 times. To evaluate the influence of posterior uncertainty on the prediction interval, the expected vital rate values as a function of the covariate given the mean posterior values of the controlling parameters were also computed.

The covariate effect "slope" parameters of these relatively simple models are summarized in Tables C.2 and their process noise variance estimates in Table C.3. The associated predicted vital rates for the models with a single covariate per vital rate are shown in Figure C.1. In general, the expected relationship between the covariate and the vital rate held. A notable exception was the model using early life stage striped bass (SB0), the direct effects on delta smelt recruitment and survival are likely only to be detrimental, but for which the modeled recruitment, post-larval and juvenile survival rates responded positively to increases in their densities. This suggests that the decline in these conspecifics along with delta smelt based on shared habitat quality during the time period studied overwhelms any direct interspecific interaction effects. Based on this, the SB0 covariate was not considered in the global models. Other predator/competitor indices were retained in the global models without prior inspection of the direction of their effects because they were hypothesized to be important for only a subset of the vital rates and could therefore not enable estimability when considered in the absence of other covariates.

Using the subset of covariates indicated in Table C.1, two global models with the same sets of covariates for each vital rate were considered: one that used external estimates of observation error CV (Scenario 1), and one that used internally estimated observation error CV (Scenario 3) along with the observation error bias and the state process parameters. Table C.4 summarizes the (non-latent state) parameter marginal posterior distributions of the global models, and Figures C.2 and C.3 show the corresponding prior and posterior distributions. Figure C.4 compares posterior means and standard deviations of latent abundance estimates between the global model fit with fixed observation error CV and the global model with estimated observation error CV. Figure C.5 illustrates how joint posterior diffusivity increases for bias parameters ψ and vital rate intercept parameters when using an observation model described by Scenario 3 compared to those of using Scenario 1.

Table C.1: Covariates considered for modeling recruitment and survival. For each covariate, the vital rates it was used for are shown in the vital rates column. Generally covariates are means or medians of daily values over Mar-May, Jun-Aug, Sep-Nov, and Dec-Feb for ρ, ϕ_{PL}, ϕ_J, and ϕ_{SA}, respectively, with a few exceptions (that shift the start and end times by one month) related to data availability or management needs. The direction column shows the expected effect. The * indicates the subset of covariates considered in the global model after removing highly collinear covariates or those that had strong effects in the opposite of their expected direction.

Predictor	Vital rates	Direction	Remarks
Inflow	ρ^*, ϕ^*_{PL}, ϕ_J, ϕ^*_{SA}	$+$	Inflow, an omnibus habitat condition measure.
Outflow	ρ, ϕ_{PL}, ϕ_J, ϕ_{SA}	$+$	Outflow, an omnibus habitat condition measure.
EI ratio	ρ, ϕ_{PL}, ϕ_J, ϕ_{SA}	$-$	Total exports to total inflow ratio.
OMR	ρ, ϕ_{PL}, ϕ_J, ϕ^*_{SA}	$+$	Old and Middle river flows.
X2	ρ^*, ϕ_{PL}, ϕ^*_J, ϕ_{SA}	$-$	Approximate location of the 2-ppt isohaline. $X2_{t-1}$ denotes the fall X2 value of the prior calendar year used for making predictions about cohort t recruitment.
LSZ	ϕ_{PL}, ϕ_J	$+$	Volume of low salinity zone habitat.
Mallard	ϕ_{PL}, ϕ_J	$-$	Salinity at Mallard Island.
Secchi	ρ^*, ϕ^*_{PL}, ϕ^*_J	$-$	Water clarity as measured by Secchi depth (cm).
South Secchi	ϕ^*_{SA}	$-$	A water clarity covariate based on Secchi data collected only from the south region of the Delta.
Temperature	ρ^*, ϕ^*_{PL}, ϕ^*_J, ϕ^*_{SA}	$-/unc$	Temperature (deg C). Cooler temperatures are expected to be better for recruitment and summer survival. Temperature effects for fall and winter survival are uncertain (unc).
ACM	ρ^*, ϕ^*_J, ϕ^*_{SA}	$+$	Large prey availability for late juveniles and adults.
NJ	ρ^*	$+$	Small prey availability for early life history fish.
NJACM	ϕ_{PL}	$+$	Combined small and large prey availability. Highly correlated with inflow in the summer.
ISS	ρ^*, ϕ^*_{PL}	$-$	Inland silverside (*Menidia beryllina*), a competitor.
SB0	ρ, ϕ_{PL}, ϕ_J, ϕ_{SA}	$-$	Juvenile striped bass (*Morone saxatilis*), a competitor.
SB1	ϕ^*_{SA}	$-$	Adult striped bass, a predator.
TFS	ρ^*, ϕ^*_{PL}	$-$	Threadfin shad (*Dorosoma petenense*), a competitor.
TG	ρ^*, ϕ^*_{PL}	$-$	Tridentiger goby (*Tridentiger sp.*), a competitor.

Table C.2: Covariate effect "slope" parameter posterior summaries for the single covariate per vital rate models. LCI and UCI are the lower and upper values of the 95% highest posterior density credible interval, respectively. Evi, the evidence, is the proportion of the posterior above zero when the expected effect is positive, proportion of the posterior below zero when the expected effect is negative; if direction is uncertain (unc), evidence is the proportion above or below zero if the mean is above or below zero, respectively. The food model uses NJ, NJACM, ACM, and ACM predictor variables for ρ, ϕ_{PL}, ϕ_J, and ϕ_{SA}, respectively.

	Recruitment				Post-larval survival				Juvenile survival				Sub-adult survival			
Model	Mean	LCI	UCI	Evi	Mean	LCI	UCI	Evi	Mean	LCI	UCI	Evi	Mean	LCI	UCI	Evi
Inflow	0.08	-0.21	0.38	0.71	1.11	0.18	2.09	0.99	-0.14	-1.01	0.69	0.36	-0.01	-0.91	0.85	0.49
Outflow	0.06	-0.24	0.35	0.65	1.31	0.24	2.50	0.99	0.12	-0.66	0.84	0.63	0.02	-0.89	0.97	0.52
EIratio	-0.23	-0.53	0.06	0.94	-0.31	-1.19	0.63	0.76	-0.22	-1.03	0.58	0.72	0.08	-0.81	1.02	0.44
X2	-0.17	-0.46	0.13	0.88	-0.96	-1.84	-0.15	0.99	0.01	-0.79	0.79	0.49	0.19	-0.76	1.13	0.34
OMR	0.12	-0.19	0.43	0.80	0.08	-0.82	1.02	0.57	0.38	-0.50	1.28	0.82	0.65	-0.19	1.48	0.95
Food	-0.06	-0.37	0.24	0.35	1.06	0.18	1.99	0.99	-0.25	-1.01	0.48	0.24	0.28	-0.63	1.27	0.73
SB0	0.16	-0.12	0.45	0.13	1.22	0.38	2.10	0.00	0.34	-0.45	1.12	0.18	-0.38	-1.27	0.46	0.83

Table C.3: Standard deviations of the process noise variance for the different single covariate per vital rate models. LCI and UCI are the lower and upper values of the 95% highest posterior density credible interval, respectively.

	Recruitment $\sigma_{P,R}$			Post-larva survival $\sigma_{P,PL}$			Juvenile survival $\sigma_{P,J}$			Sub-adult survival $\sigma_{P,SA}$		
Model	Mean	LCI	UCI	Mean	LCI	UCI	Mean	LCI	UCI	Mean	LCI	UCI
Inflow	0.54	0.31	0.77	1.41	0.67	2.38	1.68	0.97	2.55	1.57	0.75	2.60
Outflow	0.52	0.30	0.77	1.30	0.64	2.09	1.61	0.89	2.46	1.61	0.74	2.65
EIratio	0.52	0.29	0.75	1.70	0.85	2.70	1.58	0.84	2.45	1.64	0.75	2.73
X2	0.51	0.27	0.75	1.36	0.71	2.14	1.65	0.89	2.52	1.68	0.74	2.82
OMR	0.55	0.33	0.80	1.78	0.90	2.79	1.63	0.82	2.57	1.27	0.41	2.32
Food	0.54	0.31	0.79	1.43	0.76	2.24	1.62	0.93	2.45	1.52	0.70	2.51
SB0	0.50	0.28	0.73	1.16	0.58	1.82	1.52	0.85	2.31	1.60	0.79	2.65

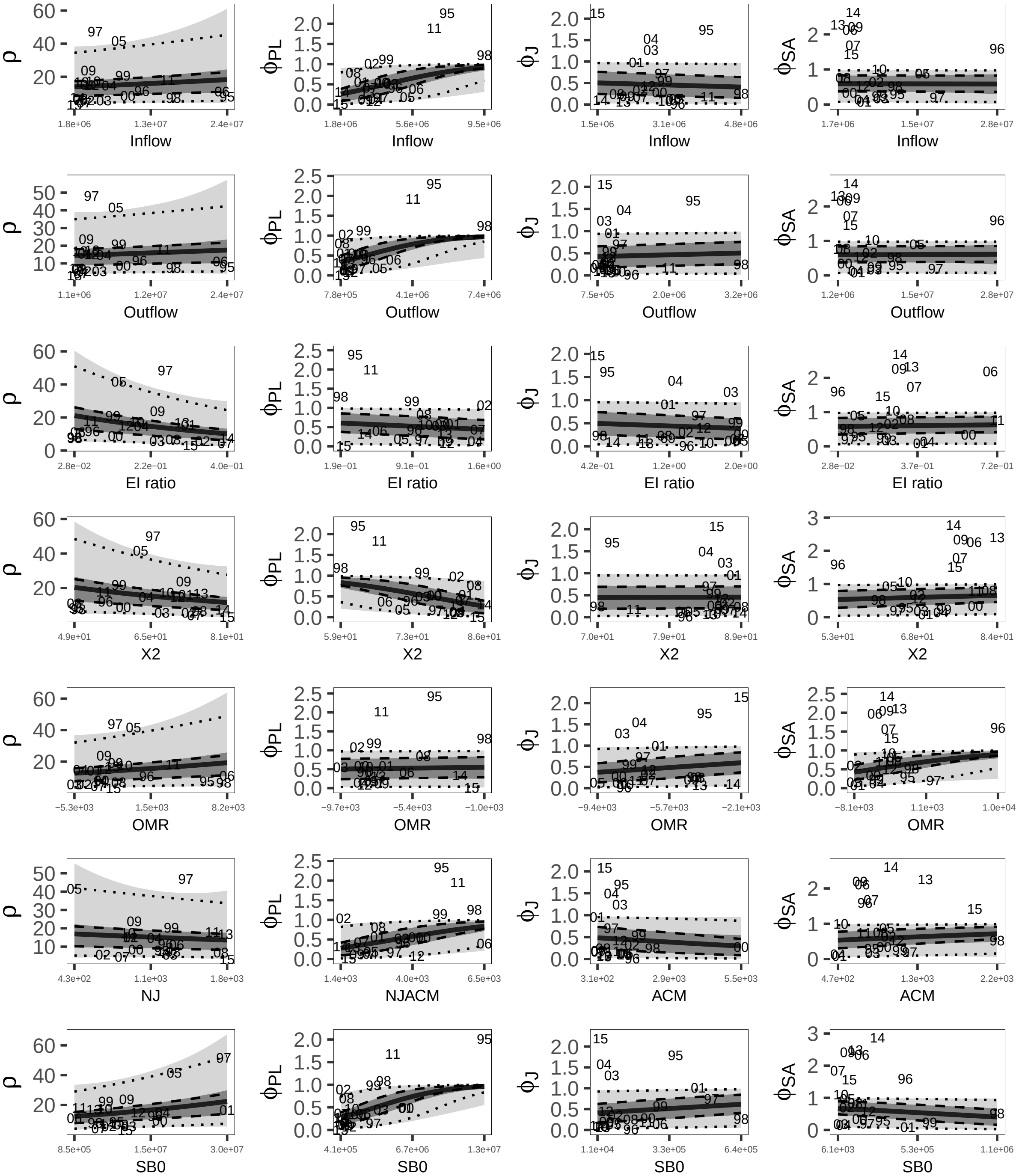

Figure C.1: Predicted vital rates for preliminary models using a single predictor variable of the same type for each state process model. Each panel row corresponds to a different model and the columns are the different vital rates. The solid curved lines show expected values, dark and light grey shadings show the $100(1-\alpha)\%$ central credible intervals for $\alpha = 0.5$ and $\alpha = 0.05$, respectively, and include posterior parameter estimate uncertainty. The dashed and dotted lines show the 50% and 95%, respectively, central credible intervals using the mean values of the posterior. Units are multi-day totals (inflow, outflow, and EI ratio), means (X2, OMR, food), or indices (SB0), over the time step of each vital rate.

Table C.4: Posterior mean, standard deviation (SD), evidence (Evi), and lower and upper 95% highest posterior density credible intervals of parameters for the global model with either fixed or estimated observation error CV. Evidence is the proportion of the posterior above zero when the expected effect is positive (Direction=pos), proportion of the posterior below zero when the expected effect is negative (Direction=neg); if Direction is uncertain (unc), evidence is the proportion above or below zero if the mean is above or below zero, respectively.

		Fixed observation error CV					Estimated observation error CV				
Parameter	Dir	Mean	SD	Evi	Lower	Upper	Mean	SD	Evi	Lower	Upper
Recruitment											
Intercept		2.53	0.16		2.21	2.84	2.33	0.20		1.93	2.68
Outflow	pos	0.07	0.20	0.63	-0.31	0.48	-0.04	0.17	0.38	-0.40	0.27
Secchi	neg	0.25	0.21	0.11	-0.16	0.66	0.25	0.21	0.13	-0.16	0.68
Temperature	neg	-0.46	0.29	0.94	-1.05	0.12	-0.35	0.27	0.90	-0.90	0.22
ACM	pos	0.18	0.22	0.80	-0.26	0.59	0.08	0.20	0.65	-0.29	0.47
NJ	pos	-0.18	0.23	0.21	-0.63	0.29	0.03	0.22	0.55	-0.42	0.38
TFS	neg	0.26	0.22	0.11	-0.17	0.71	0.30	0.20	0.07	-0.10	0.70
ISS	neg	0.05	0.23	0.42	-0.39	0.52	0.16	0.20	0.20	-0.24	0.57
TG	neg	0.28	0.25	0.13	-0.24	0.76	-0.09	0.29	0.67	-0.59	0.46
$X2_{t-1}$	neg	-0.16	0.17	0.83	-0.50	0.20	-0.03	0.19	0.56	-0.42	0.34
Post-larval survival											
Intercept		0.18	0.44		-0.68	1.04	0.04	0.61		-1.13	1.24
Outflow	pos	0.74	0.50	0.93	-0.25	1.72	0.58	0.51	0.87	-0.49	1.53
Secchi	neg	-0.47	0.43	0.87	-1.33	0.36	-0.32	0.45	0.75	-1.20	0.53
Temperature	neg	-0.25	0.45	0.72	-1.15	0.62	-0.37	0.48	0.77	-1.33	0.56
ISS	neg	-0.27	0.44	0.73	-1.12	0.61	-0.07	0.44	0.56	-0.93	0.79
TG	neg	0.10	0.48	0.41	-0.84	1.03	-0.22	0.44	0.69	-1.10	0.64
Juvenile survival											
Intercept		-0.29	0.43		-1.11	0.57	0.14	0.66		-1.00	1.40
X2	neg	0.03	0.39	0.47	-0.72	0.81	-0.13	0.40	0.63	-1.06	0.61
Secchi	neg	-0.65	0.45	0.93	-1.54	0.24	-0.45	0.52	0.81	-1.52	0.53
Temperature	unc	0.57	0.44	0.91	-0.28	1.48	0.64	0.44	0.93	-0.27	1.54
ACM	pos	-0.27	0.37	0.22	-1.01	0.47	0.06	0.38	0.53	-0.63	0.90
Sub-adult survival											
Intercept		0.58	0.34		-0.07	1.28	0.15	0.50		-0.90	1.10
Outflow	pos	-0.03	0.39	0.46	-0.85	0.72	-0.43	0.45	0.17	-1.36	0.45
OMR	pos	0.74	0.33	0.98	0.07	1.37	0.60	0.39	0.94	-0.21	1.36
South Secchi	pos	1.12	0.43	1.00	0.29	1.95	0.42	0.43	0.85	-0.40	1.35
Temperature	unc	-0.13	0.27	0.31	-0.66	0.38	0.22	0.35	0.73	-0.46	0.86
ACM	pos	0.21	0.28	0.78	-0.34	0.75	0.49	0.36	0.92	-0.17	1.23
SB1	neg	-0.22	0.24	0.83	-0.69	0.28	-0.10	0.34	0.64	-0.75	0.58
Process variance											
$\sigma_{P,R}$		0.57	0.16		0.29	0.89	0.17	0.15		0.00	0.47
$\sigma_{P,PL}$		1.42	0.41		0.70	2.22	0.75	0.48		0.00	1.62
$\sigma_{P,J}$		1.57	0.42		0.87	2.40	0.74	0.52		0.00	1.70
$\sigma_{P,SA}$		0.54	0.36		0.01	1.20	0.49	0.45		0.00	1.37
Observation error bias											
ψ_{STN}		0.42	0.07		0.30	0.57	0.51	0.19		0.21	0.86
ψ_{FMWT}		0.19	0.03		0.13	0.24	0.15	0.04		0.08	0.24
Observation error CV											
$CV[n_{PL}]_{O,In}$							0.79	0.23		0.34	1.30
$CV[n_J]_{O,In}$						0.79	0.25		0.36	1.34	
$CV[[n_{SA}]_{O,In}$							1.09	0.23		0.70	1.50
$CV[n_{A,SMWT}]_{O,In}$							0.89	0.32		0.37	1.50
$CV[n_{A,SKT}]_{O,In}$							0.34	0.24		0.01	0.79

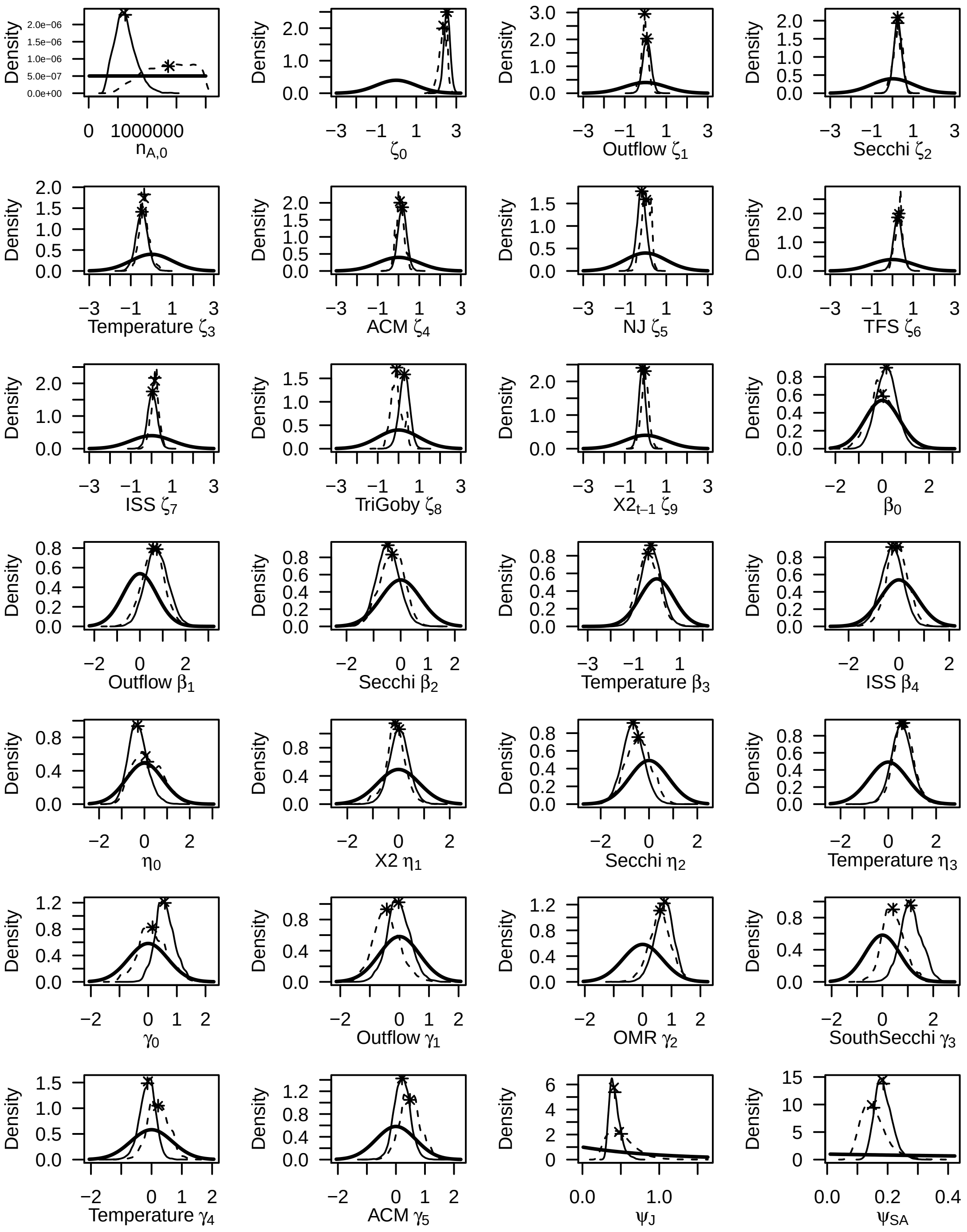

Figure C.2: Prior and posterior distributions for the initial latent abundance ($n_{A,0}$), state process parameters, and observation bias parameters. Thick line- prior; thin line- posterior from model using externally calculated observation error CV (observation model Scenario 1); dashed line- posterior from model internally estimating observation error CV (observation model Scenario 3); + is at the mean; x is at the median.

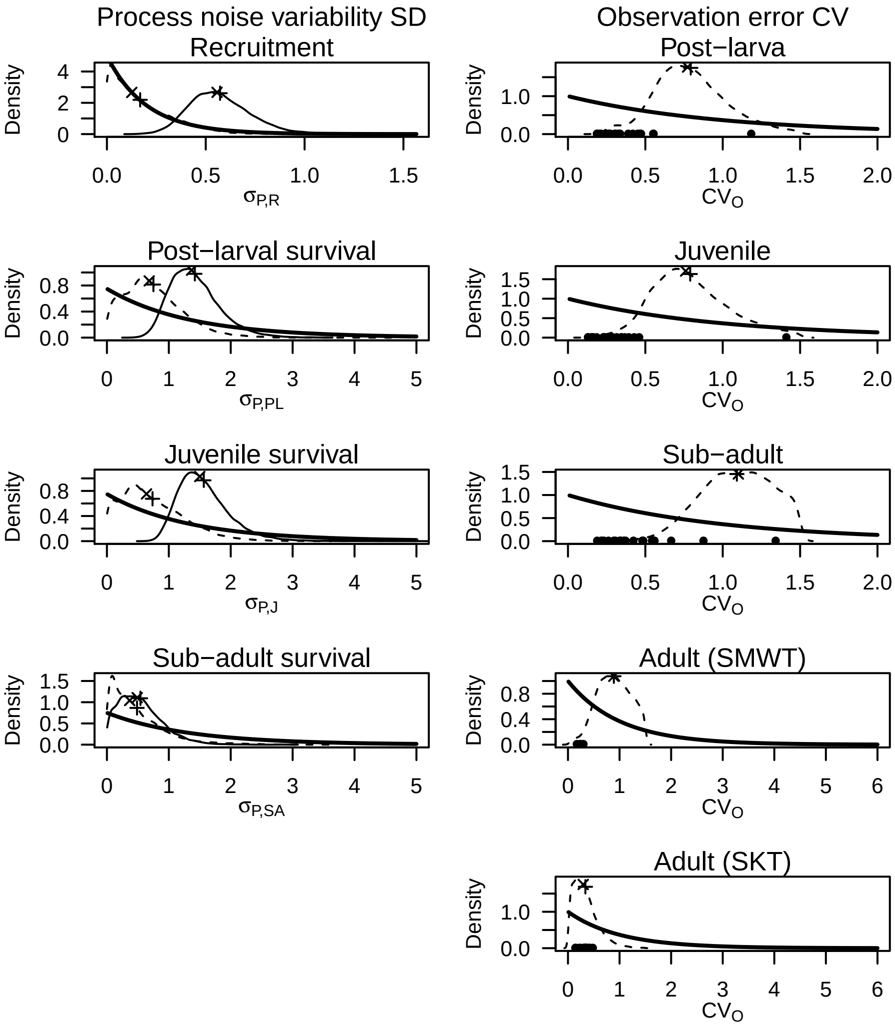

Figure C.3: Variance parameter comparisons of prior and posterior distributions. Process variance (left column) and observation error coefficient of variation (right column) from the delta smelt global models. Thick line- prior; thin line- posterior from model using externally calculated observation error CV (observation model Scenario 1); dashed line- posterior from model internally estimating observation error CV (observation model Scenario 3); + is at the mean; x is at the median; dots in the right column along the x-axis are the externally (to the SSM) estimated values.

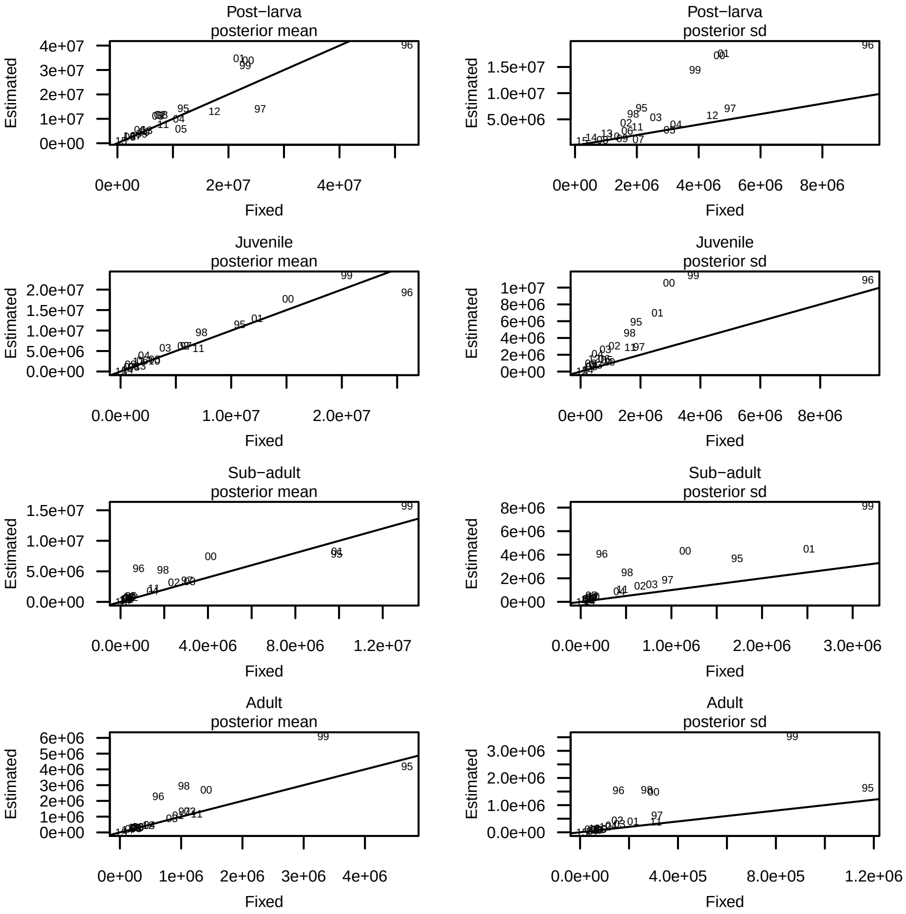

Figure C.4: Latent abundance posterior means (left column) and standard deviations (right column) based on the global model fitted with fixed observation error CV (x-axis) and estimated observation error CV (y-axis). Numbers in the plot indicate the cohort.

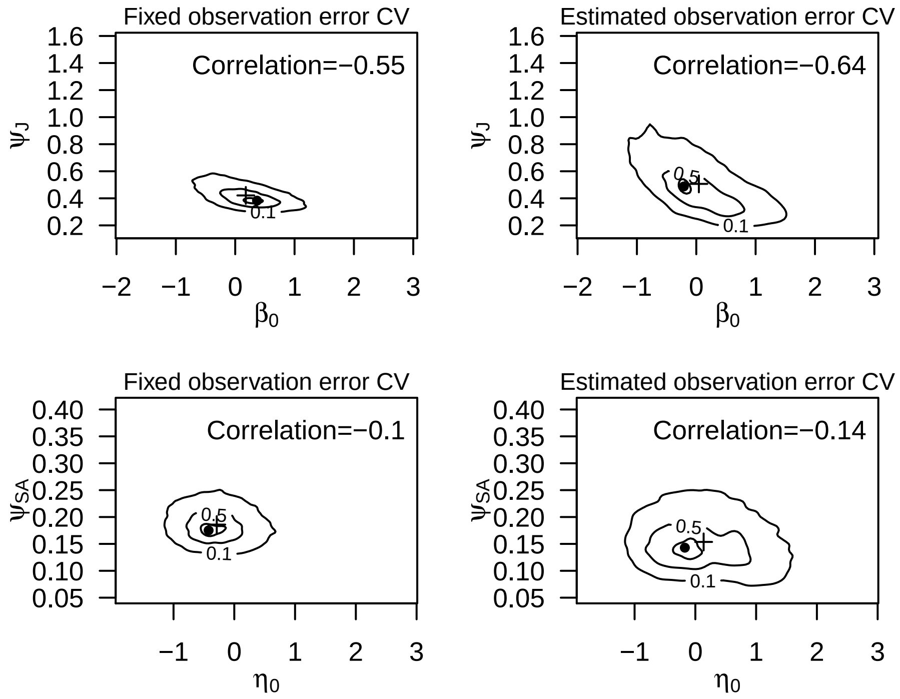

Figure C.5: Joint posterior density plots of the observation bias and the survival intercept parameters for juvenile (top row) and sub-adult (bottom row) survival and observation models. Joint density plots are scaled to lie between 0 and 1, levels are drawn at the 0.9, 0.5 and 0.1 quantiles of the distribution, the dot is at the maximum, and the cross is at the marginal mean.
